# A34 NONALCOHOLIC FATTY LIVER DISEASE AND LIVER FIBROSIS INCREASE CARDIOVASCULAR RISK IN PATIENTS WITH INFLAMMATORY BOWEL DISEASES

**DOI:** 10.1093/jcag/gwac036.034

**Published:** 2023-03-07

**Authors:** D Kablawi, F Aljohani, C S Palumbo, S Restellini, A Bitton, G Wild, W Afif, P L Latakos, T Bessissow, G Sebastiani

**Affiliations:** 1 Division of Gastroenterology and Hepatology, McGill University Health Centre; 2 Division of Gastroenterology, Jewish General Hospital, Montreal, Canada; 3 University Hospital of Geneva, Geneva, Switzerland

## Abstract

**Background:**

Non-alcoholic fatty liver disease (NAFLD) is strongly associated with cardiovascular disease in the general population. Both NAFLD and cardiovascular diseases seem more frequent in patients with inflammatory bowel disease (IBD).

**Purpose:**

We aimed to assess the effect of NAFLD and associated liver fibrosis on the cardiovascular risk in people with IBD.

**Method:**

We prospectively included IBD patients undergoing a routine screening program for NAFLD by transient elastography (TE) with associated controlled attenuation parameter (CAP). NAFLD and significant liver fibrosis were defined as CAP >275 dB/m and liver stiffness measurement (LSM) by TE ≥8 kPa, respectively. Nonalcoholic steatohepatitis (NASH) with liver fibrosis was defined as Fibroscan-aspartate aminotransferase (AST) score (FAST) >0.35. Cardiovascular risk was assessed with the atherosclerotic cardiovascular disease (ASCVD) risk estimator proposed by the American Heart Association and computed from age, sex, race, lipid pattern, blood pressure, diabetes treatment and smoking. Based on the American Heart Association guidelines, the 10-year cardiovascular risk by ASCVD was categorized as low if <5%, borderline if 5%–7.4%, intermediate if 7.5%–19.9% and high if ≥20% or if previous cardiovascular event.Predictors of intermediate-high cardiovascular risk were investigated by multivariable logistic regression analysis.

**Result(s):**

We included 405 patients with IBD (54% female; mean age 45+15 years; mean BMI 26+5 Kg/m^2^; 31% with ulcerative colitis; 7% with diabetes; 14% with hypertension). Overall, 278 (68%), 23 (6%), 47 (12%) and 57 (14%) were categorized as at low, borderline, intermediate and high ASCVD risk, respectively. NAFLD and significant liver fibrosis were found in 129 (32%) and 35 (9%) patients, respectively. NASH with fibrosis was found in 11 (3%) patients. Patients with NAFLD and with significant liver fibrosis diagnosed by TE with CAP had higher proportion of intermediate-high ASCVD risk category (see Figure). These findings were confirmed also in young IBD patients <55 years old with NAFLD. No difference in ASCVD risk was detected for FAST score. After adjusting for IBD disease activity, significant liver fibrosis and BMI, predictors of intermediate-high ASCVD risk were NAFLD (adjusted odds ratio [aOR] 2.97, 95% confidence interval [CI] 1.56–5.68), IBD duration (aOR 1.55 per 10 years, 95% CI 1.22–1.97), and ulcerative colitis (aOR 2.32, 95% CI 1.35–3.98). Only 30% of IBD patients classified as intermediate-high ASCVD risk were on statin treatment, with no difference between patients with and without NAFLD.

**Image:**

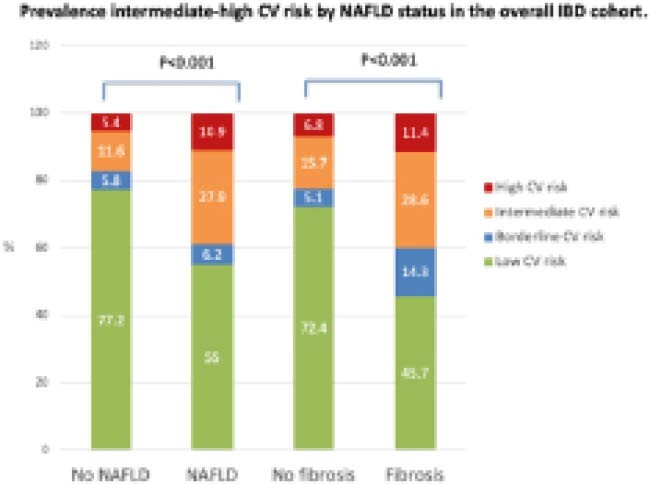

**Conclusion(s):**

NAFLD increases cardiovascular risk, independently of age, IBD-related factors and BMI.

A potential deliverable of our finding is the targeted cardiovascular assessment in IBD patients with NAFLD and appropriate initiation of statin, particularly if they have longer IBD duration and ulcerative colitis.

**Please acknowledge all funding agencies by checking the applicable boxes below:**

None

**Disclosure of Interest:**

None Declared

